# Evolving trends and challenges in sustainable architectural design; a practice perspective

**DOI:** 10.1016/j.heliyon.2024.e39400

**Published:** 2024-10-16

**Authors:** Emeka J. Mba, Francis O. Okeke, Ajuluchukwu E. Igwe, Chinelo A. Ozigbo, Peter I. Oforji, Ikechukwu W. Ozigbo

**Affiliations:** a Department of Architecture, University of Nigeria, Enugu Campus, Enugu State, Nigeria; b School of Engineering, Technology and Design, Canterbury Christ Church University, Kent, UK; c Department of Estate Management, University of Nigeria, Enugu Campus, Enugu State, Nigeria

**Keywords:** Sustainable architecture, Architectural practice, Sustainability, Design, Built environment

## Abstract

Architects play a crucial role in shaping sustainable built environments that balance utility and aesthetics without compromising ecological integrity. Hence embracing sustainability remains an urgent imperative as construction activities expand to meet Nigeria's development needs. This research examines how architects in Enugu Metropolis, Nigeria are assimilating principles of sustainability within their design practices against a transforming professional backdrop. The study utilized primary data targeting 126 registered architectural firms in the study area through a questionnaire survey and achieved 88.9 % response rate. Data were analyzed using JASP and Pearson product moment correlation. Results revealed high conceptual awareness of sustainability among architects, with 32.2 % consistently applying strategies like renewable energy systems in designs. Economic considerations and supportive regulations emerged as key assimilation motivators. However, uneven integration levels underline lingering barriers. While 40.6 % rated sustainability incorporation in education as very good, 13.5 % perceived it as moderate, indicating gaps in practical competencies. Statistical analysis (P-value = 0.001) showed a significant positive relationship between architects' awareness of sustainability concepts and their implementation of sustainable design practices. These findings highlight the need for strengthened educational programs, policy incentives, and industry collaborations to accelerate sustainable architecture adoption. Recommendations include updating curricula, mandating green ratings for public projects, incentivizing developers, and promoting interdisciplinary knowledge transfer. By addressing identified gaps, it implies that architects can lead the sustainability transition while meeting local built environment needs, ensuring a more climate-compatible and socially equitable habitat creation.

## 1 Introduction

The unprecedented growth in global population and rapid urbanization have created mounting pressures on the architecture and construction sector to support sustainable development through resilient and eco-friendly buildings and infrastructure [1]. Population rise especially in emerging regions coupled with accelerating rural-urban migration to metropolitan hubs, has led to expanding resource demands and sustainability challenges [2,3]. Projections indicate that along with demographic shifts, the imperative for smarter green buildings and upgraded technologies will intensify given the building and construction sector's sizable carbon footprint [4,5]. As architect and designers are entrusted with the responsibility to create spaces that leave a positive legacy in the built environment [6]; the architectural and construction firms invariably face escalating societal expectations to enable greener habitats and improved quality of life through productive solutions balancing growth ambitions with ecological consciousness. This backdrop informs the rationale for assessing how architects have assimilated sustainable best practices and continually enhance skills to deliver positive societal change.

The increasing global focus on achieving net zero energy consumption and reducing carbon emissions across major sectors has put conventional buildings under scrutiny for their environmental impact [7]. As a result, the demand for sustainable buildings has gained momentum, driven by the growing awareness of the negative environmental consequences associated with traditional construction practices [8]. Sustainability in the built environment, particularly through the concept of sustainable buildings, has become a central topic of discussion due to the substantial financial, societal, and environmental implications of the building and construction industry [9]. This industry, along with the policy standards that regulate it, influence the future of our planet. Conventional buildings are often criticized for their high energy consumption, reliance on fossil fuels, and significant contribution to greenhouse gas emissions. They also tend to use materials that have a high embodied energy, meaning that a considerable amount of energy is required for their production, transportation, and disposal [10]. Additionally, traditional construction methods can lead to waste generation, depletion of natural resources, and negative impacts on biodiversity. The shift towards sustainable buildings is not only driven by environmental concerns but also by the potential economic benefits. Sustainable buildings can lead to lower operating costs, increased property values, and improved employee productivity. Furthermore, as governments and organizations set ambitious targets for carbon reduction and energy efficiency, the demand for sustainable buildings is expected to grow, creating new opportunities for the construction industry. However, the transition to sustainable buildings is not without challenges. It requires significant changes in the way buildings are designed, constructed, and operated, as well as the adoption of new technologies and materials.

Architecture as a profession does not only revolve around creating shelter and spaces where humans carry out their daily activities rather, it extends to the point of influencing the way we live and our daily activities as well as ecosystem functions [11]. One significant aspect to consider is the evolution of architectural practices in response to environmental challenges and societal demands for more sustainable built forms. As highlighted by Smith [12], architects in recent times have increasingly embraced sustainability principles in their designs, moving beyond conventional approaches to integrate green building technologies, passive design strategies, and renewable energy systems to minimize environmental impacts. With buildings being the major contributors to the release of greenhouse gases into the atmosphere, architects as one of the major players in the construction industry are charged with the duty of addressing these issues, by not only designing for aesthetics but by also applying sustainable design principles into their designs as this will help to create a built environment that mitigates environmental impact. Sustainability according to McGraw-Hill [13] is described as the long-term viability of a community, a set of social institutions, or a societal practice that embodies an intergenerational ethical principle, where the environmental and economic actions undertaken by present individuals aim to ensure that future generations have similar opportunities to enjoy wealth, utility, or welfare. Sustainable architecture integrates principles of energy efficiency, resource conservation, site selection, passive design strategies, renewable energy sources, and the well-being of the occupants into the design process [14].

Though prior studies have assessed sustainable building adoption barriers [[15], [16], [17]] and benefits in Nigeria [18], a specific spotlight on architects' dynamic responsibilities around sustainability amid industry transformations remains lacking. Nnaemeka-okeke et al. [19] examined the role of the architect in achieving the 2030 agenda for sustainable development in Nigeria and provided useful insight on diffusing the architect role into the SDGs, evidencing growing awareness levels among construction professionals [20]. Yet, principle integration by architects at project conception stages warrants dedicated scrutiny being prime shapers of space usage, materials and operational impacts. Building and construction operation in Nigeria contribute significantly environmental pollution and degradation [21], necessitating mitigation efforts by decision-makers like architects. As Ajayi et al. [22] noted, the country's largely traditional construction approaches constrain integration of sustainability considerations in project lifecycles. Factors like persisting capability gaps [23], gaps in specialized knowledge, regulatory limitations, and client preferences stall mainstreaming of green building expertise by key decision-makers like architects. This can generate disconnects between industry outlooks and realities surrounding sustainability commitments. Therefore, a granular investigation is imperative through collection of empirical data because the previous systematic review study of [24] analyzing the role of architects in achieving the sustainable cities goal by 2030 did not address the gap. Moreover, while research of Obi et al. [25] recognizes sustainability coverage in construction curricula, rapidly evolving technologies, policies, and best practices make continuous professional development for practitioners indispensable. But limited assessments exist interrogating alignment of academic exposure and practical workflows surrounding green integration in building life cycles.

In essence, despite advancements, gaps in understanding architectural community preparedness for sustainability commitments, institutional capacities, and barriers to field implementation in Nigeria underline the value addition of dedicated sustainability-focused role evaluations. While the study of [26] acknowledges the pivotal change agent position of architects for green transition, few studies evaluate translated actions on-ground, deepening this research need [16]. Moreover, aspects like adeptness for shifting regulations, client engagement abilities, upskilling capacities, and technology adoption readiness warrant examination. This backdrop underscores the value of dedicated urban-focused research probing formal sustainability commitments and designers' preparatory realities. Although, Ibiyeye et al. [27] have studied awareness of building sustainability among architectural student within Kaduna State University in Northwestern Nigeria. An Enugu-centered exploration can enrich domain understanding while enabling generalizability. The choice of Enugu Metropolis as the focus of this study is significant for several reasons. As a rapidly growing urban center and former eastern region capital city, with highest concentration of architects in southeastern Nigeria, Enugu faces increasing pressure on its built environment and natural resources [28]. The city's architectural landscape is undergoing significant transformation, making it an ideal case study for examining the integration of sustainable practices. Moreover, Enugu serves as a microcosm of the challenges and opportunities faced by mid-sized Nigerian cities in adopting sustainable architecture. Hence, this study will provide insights that can be potentially applicable to similar urban contexts across Nigeria and sub-Saharan Africa. Therefore, the present research aims to investigate the evolving roles and practices of architects in integrating principles of sustainability into architectural design within the specific context of Enugu Metropolis, Nigeria. The following objectives were pursued to achieve the aim above:

• To analyze the extent to which architects in Enugu metropolis are incorporating sustainable design principles, with a focus on resource efficiency and renewable energy integration, and the impact of these practices on the local built environment
• To identify key factors influencing the adaptation of architects to sustainable practices, encompassing changes in design methodologies and integration of environmental considerations.
• To examine the incorporation of sustainable design principles into architectural education, addressing how architects are being prepared for the challenges and opportunities in sustainable practice.

The hypothesis [Ho] raised was that; there is no significant relationship between awareness of sustainable design concept, principles, and SGDs in architecture and architectural design practice in the study area. This analysis is beneficial as it provides a basic knowledge of the prevailing practice situation, serving as reference material for further research and a guide for policy formulation. As principal overseers coordinating diverse stakeholders throughout a building's lifecycle, the architectural fraternity's preparedness and leadership hold profound influence over the trajectory and outcomes of development projects across communities. Exploring architects' evolving job roles and preparedness levels against the testing backdrop of proliferating populations amidst climate change therefore offers timely insights into how this vital profession can drive broad-based progress. Findings can enrich domain understanding while enabling targeted interventions to elevate sustainability assimilation within architectural work guiding the city-region's upcoming built terrain.

## 2 Literature review

### 2.1 Historical background and concept of sustainable architectural design

**Fig. 1:**
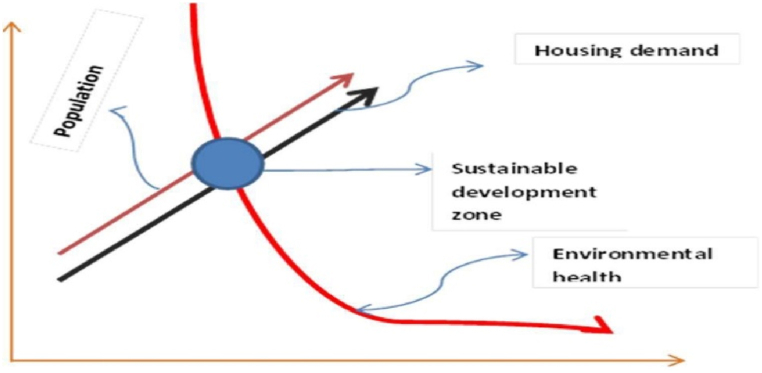
Balance between demand for buildings and environmental health.

**Source:** Ibok et al., 2019

Architectural discourse over the past 120 years reflects reactions to industrialization's economic and ecological crises [29]. This era has seen the evolution of architectural practices and philosophies in response to the multifaceted challenges posed by rapid industrialization, urbanization, and their accompanying environmental impacts. Early sustainable buildings tended towards rationalist, functionalist designs, striving to blend utility with an appreciation for natural beauty. However, the concepts of bioclimatic adaptation, hygiene, and empirical evidence remained largely undeveloped until the mid-20th century. The 1950s marked a significant turning point with the pioneering work of the Olgyay brothers, who established architecture's first research lab. Their efforts laid the groundwork for a methodological approach to sustainable design, firmly rooted in scientific principles [30]. This breakthrough enabled architects to incorporate climate-responsive strategies and environmental data into their designs, fostering a deeper understanding of the interplay between buildings and their natural surroundings. Today, amidst intensifying climate change emergencies and continuously evolving environmental laws, architects face an urgent, heightened responsibility to advance sustainable building practices. The construction industry is a major contributor to environmental degradation, and the need to mitigate its impact is more pressing than ever [31]. Consequently, sustainability has become an indispensable element of contemporary architectural design. Sustainable architectural design seeks to holistically integrate aesthetic, economic, social, and ecological considerations, aiming to create buildings that are not only functional and beautiful but also environmentally responsible. As the global population continues to rise, the demand for additional shelter grows, exacerbating environmental pressures through increased development [32]. This surge in building activity underscores the necessity for sustainable practices that can balance human needs with the preservation of environmental health. Ibok et al. [33] conceptualized this delicate relationship, illustrating the balance between meeting the demand for buildings and maintaining ecological integrity (see Fig. 1). Their work emphasizes the importance of global collective action in addressing these challenges.

However, the pathway to sustainable architecture is fraught with challenges. Okeke et al. [34] argued that sustainable architecture presents a viable solution to various pressing issues, including environmental crises, economic growth, resource depletion, ecosystem damage, and biodiversity loss. By adopting sustainable design principles, architects can contribute to mitigating these problems, fostering a built environment that supports both human and environmental well-being.

### 2.2 Principles of sustainable architecture

• **Energy use efficiency**: Energy efficiency in sustainable architecture involves maximizing the use of natural resources to reduce dependence on artificial energy sources. This approach involves optimizing the use of daylight to provide natural illumination, reducing the reliance on artificial lighting and thus lowering electricity consumption. Similarly natural ventilation strategies can be employed to minimize the demand for mechanical air conditioning systems [35]. Innovative uses of rainwater, such as rainwater harvesting systems, can also contribute to sustainable domestic water use. These principles are particularly relevant in tropical climates like Indonesia, where natural ventilation and daylighting can be optimized effectively.
• **Land use efficiency**: Efficient land use in sustainable architecture ensures that not all available land is covered with buildings, allowing for green spaces and parks to thrive. This involves designing compact and integrated building layouts that maximize green potential through innovations such as roof gardens, hanging gardens, and living walls. Preserving existing plants and trees on-site is crucial, as they can be integrated into the design to enhance the ecological value and aesthetic appeal of the space. Open designs that connect interior spaces with gardens further promote flexibility and a seamless interaction with nature [36].
• **Material use efficiency**: Material efficiency in sustainable architecture focuses on reducing waste by repurposing leftover and used materials. For instance, residual wood from construction projects can be repurposed for use in other areas of the building, while materials from demolished structures can be recovered and reused. Also, prioritizing the use of materials that are abundant and renewable, such as bamboo, helps in conserving scarce resources. This approach not only minimizes waste but also promotes the use of sustainable materials in construction.
• **Using new technologies and materials**: Leveraging innovative technologies and materials promote sustainability in architecture. Utilizing renewable energy sources like solar, wind, and hydropower can enable establishments and homes self-sufficiently generate their own electricity [37]. The discovery and application of new materials on a global scale offer possibilities for integrating renewable resources that are both affordable and available for innovation. For instance, materials such as bamboo, known for its rapid growth and versatility, present opportunities for sustainable construction.
• **Waste management:** Recent advancements have shifted towards developing closed-loop systems aimed at maximizing resource recovery. One promising approach is the establishment of decentralized domestic waste treatment systems that effectively handle greywater and blackwater, alleviating pressure on municipal water infrastructure. According to He et al. [38], innovative strategies worth exploring include organic waste decomposition systems that promote natural breakdown in situ, as well as transforming common objects into materials that can either be recycled or decompose easily in the environment. These initiatives not only contribute to sustainability but also foster a more circular economy within architectural practices.

### 2.3 Sustainable architecture design strategies

In the quest of environmentally responsible habitat, environmental professionals have developed a diverse range of innovative design methodologies. These approaches not only aim to minimize the ecological footprint of buildings but also enhance functionality and occupant well-being. The emergence of eco-conscious design reflects a broader societal shift toward sustainability, where the built environment is harmoniously integrated with natural ecosystems. When planning and designing a building, there are numerous strategies available as references for sustainable architectural design. Below are some key strategies that have become central to eco-conscious architectural practices:

• **Climate-responsive design:** One of the foremost strategies employed by architects is climate-responsive design. This approach prioritizes building orientation and envelope design to maximize the use of natural light and ventilation, thereby reducing reliance on artificial systems. By strategically positioning windows and employing overhangs or shading devices, architects can significantly lower energy consumption [1]. Such designs leverage local climate conditions, enhancing thermal comfort and reducing heating and cooling demands. This not only contributes to energy efficiency but also promotes a healthier indoor environment for occupants.
• **Resource efficiency:** Another critical element is resource efficiency. The integration of cutting-edge technologies such as smart lighting systems, advanced HVAC technologies, and high-performance insulation materials optimizes energy use and reduces operational costs. These innovations allow buildings to respond dynamically to occupancy levels and environmental conditions, further minimizing energy waste. Resource efficiency also extends to water use; low-flow fixtures and greywater recycling systems help conserve water and reduce the environmental impact of new constructions.
• **Eco-friendly material selection:** This plays a vital role in sustainable design. There is a growing emphasis on using locally sourced, renewable, and low-impact materials to decrease the overall environmental burden of construction [8]. By choosing materials that require less energy to produce and transport, architects can lower the carbon footprint of their projects. Sustainable materials often include reclaimed wood, recycled metals, and natural fibres, which not only contribute to environmental sustainability but also add aesthetic value to the design.
• **Hydrological considerations:** Innovative water management systems have become standard in sustainable architectural design. Strategies like rainwater harvesting, permeable pavements, and green roofs allow for effective management of stormwater runoff, reducing the burden on municipal systems. These systems not only conserve water but also improve the surrounding landscape by promoting biodiversity and creating habitats for urban wildlife.
• **Urban ecology integration:** Incorporating living elements into architectural designs is increasingly recognized as essential for enhancing urban environments. Features such as green roofs, living walls, and urban gardens improve air quality, enhance biodiversity, and provide recreational spaces for residents. These elements not only mitigate the heat island effect often found in urban areas but also foster a sense of community by connecting inhabitants with nature.
• **Circular economy principles:** In architectural design this principle is now gaining traction. Designers are considering the entire lifecycle of buildings, focusing on adaptability, ease of deconstruction, and material reuse. By planning for the end of a building's life from the outset, architects can ensure that materials are repurposed rather than discarded, thus minimizing waste and conserving resources.
• **Existing structure utilization:** Repurposing old buildings is a trend that not only preserves cultural heritage but also significantly reduces resource consumption compared to new construction. Adaptive reuse of structures can breathe new life into historical sites while minimizing the environmental impact associated with demolition and new material production.
• **Carbon-Conscious planning:** Architects are increasingly focused on minimizing carbon emissions through renewable energy integration and efficient transportation planning. Incorporating solar panels, wind turbines, and electric vehicle charging stations into designs encourages sustainable energy use and promotes a low-carbon lifestyle among occupants.
• **Participatory design:** Engaging local communities in the design process ensures that projects meet actual needs and foster a sense of ownership. Participatory design approaches help architects understand community values, leading to more successful and accepted projects that enhance social sustainability.
• **Nature-inspired interiors:** The integration of biophilic design elements such as natural materials, interior ornamental plants, and passive lighting has been shown to improve occupant well-being and productivity. By connecting indoor spaces with nature, architects can create environments that are not only aesthetically pleasing but also psychologically beneficial.
• **Holistic impact assessment:** A comprehensive analysis of a building's environmental impact throughout its lifespan is becoming crucial in the design process. This includes evaluating energy use, water consumption, and material sourcing to ensure that the overall ecological footprint is minimized.
• **Universal design principles:** Creating spaces that are accessible and useable by people of all abilities is increasingly recognized as a key aspect of social sustainability. Universal design principles ensure that environments accommodate diverse user needs, promoting inclusivity and equity.
• **Ethical sourcing practices:** There is a growing awareness of the need to ensure that materials and products come from sources that uphold fair labour and environmental standards. Ethical sourcing not only supports sustainability but also aligns with the values of socially conscious consumers.
• **Sustainability education:** Architects are increasingly taking on the role of educators, informing clients and users about the sustainable features of their designs and how to use them effectively. This educational aspect is essential for maximizing the benefits of eco-conscious design.
• **Enduring design philosophy:** creating timeless designs that remain functional and appealing over long periods is seen as a way to reduce waste and resist throwaway culture. This enduring design philosophy encourages quality over quantity and fosters a more sustainable relationship with our built environment.

Although the pursuit of sustainable architecture is filled with exciting opportunities, the journey toward its implementation is often complicated by a range of challenges. According to Greenfield [39] designers frequently find themselves at a crossroads when trying to reconcile sustainability with the aesthetic preferences of their clients and the communities they serve. Many clients arrive with a vision shaped by conventional design standards, which can sometimes clash with eco-friendly innovations. The challenge lies in creatively integrating sustainable features and systems into visually appealing designs that satisfy both form and function. Another significant hurdle involves justifying the higher upfront costs associated with sustainable building practices; as many eco-friendly materials and technologies require a greater initial investment, leading architects to navigate the delicate task of persuading clients of their long-term benefits. This often involves presenting comprehensive analyses that highlight potential savings on energy and maintenance, as well as increased property value over time. Convincing clients that these investments are not merely expenses but rather steps toward a more sustainable future requires a blend of analytical skills and empathetic communication. Limited availability of certain eco-friendly materials can also complicate the design process. Architects may encounter difficulties in sourcing specific sustainable options, leading to delays or increased costs that can frustrate project timelines. The agility in sourcing materials calls for an in-depth understanding of the marketplace and a willingness to innovate within constraints. Also, client interest in sustainable features can vary widely, posing another challenge for architects. While some clients are enthusiastic about integrating eco-friendly elements into their designs, others may view them as secondary or even unnecessary. Architects must navigate these differing levels of interest by tailoring their proposals to align with each client's values and aspirations. This could involve educating clients about the benefits of sustainable practices and helping them envision how these features can enhance their projects, both in terms of functionality and aesthetic appeal. In addition, the rapidly evolving landscape of sustainability regulations adds another layer of complexity. Architects needs to stay informed about new codes and standards to ensure compliance and take advantage of potential incentives or certifications. This requires a commitment to ongoing professional development and engagement with industry trends, which can be demanding in an ever-changing field.

To effectively overcome these hurdles, the professional architect should foster interdisciplinary collaboration, drawing insights from engineers, environmental scientists, and urban planners. This collaborative approach often yields innovative solutions that align with sustainability goals while addressing practical challenges. Staying abreast of technological advancements is equally vital; as new materials and techniques emerge, architects can incorporate cutting-edge solutions into their designs, enhancing both performance and sustainability.

### 2.4 Incorporation of sustainable architecture into architectural education

Architectural education has progressively transformed over the past 50 years to integrate principles of sustainability, reflecting growing environmental awareness. Initial introduction as specialized courses in the 1980s has led to widespread incorporation of sustainability into ethical codes, curricula, and global collaborative initiatives like the 2030 Challenge [40]. Spurred by the UN's 1987 sustainable development definition and the Higher Education Sustainability Initiative, interdisciplinary, holistic education prepares 21st century architects for complex sustainability challenges [41,42]. Tracing back to early environmental consciousness, this educational evolution mirrors recognition of architects' pivotal role in manifesting a sustainable, resilient built environment. The integration of sustainable architecture into educational curricula is evident in various institutions, reflecting a concerted effort to equip future architects with the necessary knowledge and skills. Key considerations include foundational knowledge in sustainable concepts, specialized design studios, interdisciplinary collaboration, technology integration, case studies, understanding of codes and standards, field trips/workshops, ethical discussions, continuous evaluation, and feedback. These efforts aim to prepare graduates to contribute to environmentally conscious built environments, emphasizing interdisciplinary collaboration and innovative pedagogies. Despite challenges, ongoing initiatives are reshaping architectural education to produce professionals capable of addressing sustainable design complexities.

### 2.5 Challenges architects face in their roles in sustainable architecture

The evolving roles of architects in sustainable architecture bring forth numerous challenges, highlighting the complexity of merging environmental concerns with architectural practice. Sustainable architecture is not just about creating environmentally friendly buildings but also about balancing human needs for shelter with the imperative to preserve environmental health. This delicate balancing act presents a multifaceted array of challenges that architects must navigate to integrate sustainability effectively into their work. One primary challenge is the need for comprehensive sustainability education in architectural programs, as traditional curricula often lack sufficient coverage of sustainable design principles and practices [43]. Resistance to change within the profession further complicates this, as established practices can be difficult to alter [44]. Financial constraints also pose a substantial challenge, as sustainable materials and technologies can be more expensive upfront, deterring clients and developers. Architects must advocate for sustainable design by demonstrating long-term cost savings, health benefits, and potential property value increases, while exploring creative financing solutions. Navigating intricate regulatory frameworks, which vary widely, requires architects to stay informed about the latest regulations and ensure compliance [45]. Collaboration with diverse disciplines, including engineers, ecologists, urban planners, and sociologists, is essential but challenging, necessitating strong project management skills and a collaborative culture. Limited integration of sustainable technologies, due to a lack of awareness, perceived risks, or insufficient technical support, requires architects to stay updated on advancements and build confidence in these technologies among clients and contractors [46]. Shaping public perception is also crucial, as many may not fully understand the benefits of sustainable design. Architects must engage in public education and advocacy, showcasing successful projects and communicating broader impacts. Cultural variations add another layer of complexity, requiring sensitivity to local cultures, climates, and traditions to adapt sustainable principles effectively [47]. Post-occupancy evaluation deficiencies further complicate the landscape, as thorough evaluations are crucial but often neglected. Finally, continuous professional development is essential in this constantly evolving field. Addressing these challenges is vital for embedding sustainable principles into architectural practice, fostering a built environment aligned with sustainability goals, and creating a more sustainable future.

### 2.6 Conceptual framework and theoretical underpinnings

Sustainability in architecture encompasses a broad spectrum of practices aimed at minimizing the negative environmental impact of buildings while maximizing their positive social and economic effects. For this study, we conceptualize sustainable architectural practices as design and construction approaches that:

• Minimize resource consumption and environmental degradation
• Maximize energy efficiency and use of renewable resources
• Enhance occupant health and well-being
• Contribute positively to local communities and economies

This conceptualization aligns with the triple bottom line approach to sustainability, which emphasizes environmental, social, and economic dimensions [48]. The triple bottom line approach underscores that true sustainability cannot be achieved unless environmental protection, social equity, and economic viability are all addressed in a balanced manner. Several theories guide the adoption of sustainable architectural practices, each offering a unique perspective on how sustainability can be integrated into architectural practice:

• Diffusion of Innovation Theory [49]: This theory helps explain how sustainable architectural practices spread through professional communities. It suggests that adoption occurs in stages, with innovators and early adopters paving the way for wider acceptance. According to this theory, the rate of adoption is influenced by the perceived benefits of the innovation, compatibility with existing values, simplicity of integration, trialability, and observable results.
• Theory of Planned Behaviour [50]: This theory posits that an individual's intention to adopt sustainable practices is influenced by their attitudes toward the behaviour, subjective norms, and perceived behavioural control. This is particularly relevant in understanding architects' decision-making processes regarding sustainability. Attitudes reflect the favorable or unfavourable evaluation of the behaviour; subjective norms involve perceived social pressure to perform or not perform the behaviour; and perceived behavioral control refers to the perceived ease or difficulty of performing the behaviour.
• Ecological Modernization Theory [51]: This theory proposes that economic development and environmental protection can be mutually reinforcing. In the context of architecture, it suggests that sustainable practices can drive innovation and economic growth, leading to new markets, job creation, and technological advancements. This theory highlights the role of technological innovation and policy reforms in achieving sustainability.
• Social Practice Theory [52]: This theory emphasizes the importance of routines, habits, and social norms in shaping behaviour. It is useful for understanding how sustainable practices become embedded in architectural firms' daily operations. Social Practice Theory looks at the interplay between materials, competences, and meanings to understand how practices evolve and stabilize over time.
• Transition Theory [53]: This theory examines how socio-technical systems transition from one state to another. It is relevant for understanding the broader systemic changes required for the widespread adoption of sustainable architecture. Transition Theory focuses on multi-level perspectives involving niche innovations, socio-technical regimes, and landscape developments to explain how systemic change occurs.

These theories provide a framework for understanding the complex factors influencing the adoption of sustainable architectural practices in Nigeria. This study draws particularly on these theories to interpret architects' awareness, attitudes, and behaviours regarding sustainability. By framing our research within these theoretical perspectives, the study aim to contribute not only to the empirical understanding of sustainable architectural practices in Nigeria but also to the broader theoretical discourse on sustainability transitions in developing countries.

## 3 Research methodology

### 3.1 Study area

**Fig. 2:**
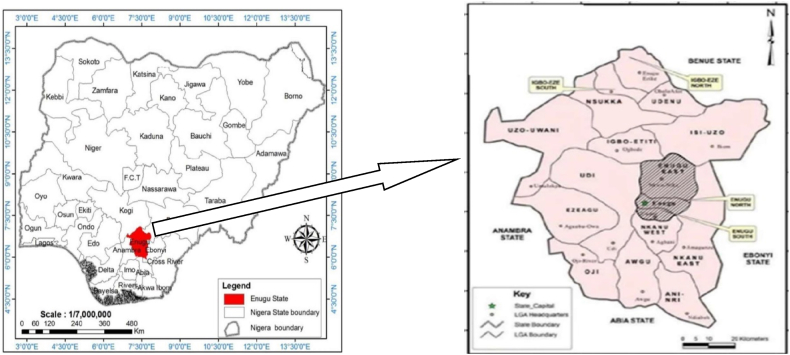
Map showing Nigeria, highlighting Enugu State.

Enugu is the capital city of Enugu State in southeast Nigeria. It lies approximately between the latitude 6° 27′ 10″ North of the Equator and 7° 30′ 40″ East of the Greenwich Meridian (see Fig. 2). It comprises several layouts which collectively link to form a grid patterned city [54]. The city is situated at an elevation that contributes to its cooler climate compared to lower-lying areas. Surrounding landscape features undulating terrain, with hills providing natural landmarks and scenic views. Enugu have a climate typical of the West African region, characterized by relatively moderate temperatures ranging from 24 °C to 30.8 °C. The area is dominated by two main seasons: the dry season and the rainy season. Additionally, a brief harmattan period around January or February temporarily disrupts the high humidity, bringing cool, dry winds from the Sahara desert, which results in a dusty atmosphere. The city has experienced a 2.3 % annual population change from 2006 to 2022, highlighting its dynamic demographic landscape. Enugu's cultural and economic vibrancy is reflected in its diverse communities, making it a significant and resilient center of growth in Nigeria [55].

The focused target population of the study consists of three main local governments which are; Enugu East, Enugu North and Enugu South. This is generally contextualized as the main urban area of Enugu state, shown in previous studies Okeke et al. [56]. This choice of Enugu Municipal as a case study stems from the fact that the city area offers a rich and multifaceted environment for research, particularly in fields such as urban studies, sociology, geography, or environmental science. Its historical significance, economic activities, and cultural diversity provide a compelling backdrop for various research. That is why authors like [57,58] have evidenced Enugu metropolis as a sample for broader generalizations. Enugu metropolis is experiencing extensive infrastructure growth accompanying urbanization and industrialization policies to solidify its regional economic hub status [59]. Mass housing schemes, commercial establishments and transport upgrades currently shape planning priorities. These will expand construction activity and resource footprints, though sustainability consciousness remains uneven [60]. Therefore, assessing alignment of architect practices with sustainability objectives is prudent.

### 3.2 Research design

This quantitative study investigates the evolving integration of sustainability in architectural practice using a survey methodology. This method was considered the most appropriate for the study, as it offers insights from professionals in the field of architecture, allowing for the collection of consistent responses from a sample of respondents [61]. Architectural firms in Enugu, Nigeria serve as the target population for standardized data gathering and statistical analysis of trends regarding sustainable design principles and education. Out of 126 registered architectural firms in Enugu state per the Architects Registration Council of Nigeria (ARCON) 2017 register, 108 comprise the sampled respondents. Stratified random sampling technique was employed to ensure representation across the different local government areas (LGAs) in Enugu, Nigeria. The total population of architectural firms was divided into three strata corresponding to the three LGAs: Enugu North, Enugu East, and Enugu South. Within each stratum, random sampling was conducted to select the respondents. A list of all architectural firms in each LGA was obtained, and firms were randomly selected using a random number generator to ensure unbiased selection. This approach ensured that the sample reflected the diversity of architectural practices in the study area. A structured questionnaire was developed based on an extensive literature review and the study objectives. The questionnaire consisted of two main sections: Socio-demographic information of respondents and the three key research focus: a) Awareness and incorporation of sustainable design principles, b) Factors enabling adaptation to sustainable practices, c) Integration of sustainability in architectural education. The instrument utilizes Likert-type scale response formats validated through expert review made up of a panel of five professionals: two senior academics with expertise in sustainable architecture, two practicing architects with over 15 years of experience in sustainable design, and one environmental policy expert. The experts assessed the questionnaire for content validity, clarity, and relevance to the research objectives. Data collection was conducted through in-person distribution of paper questionnaires to the architectural firms. This method was chosen to maximize the response rate and allow for clarification of any questions respondents might have. The data collection period spanned four weeks. Informed consent was obtained from all participants, and ethical considerations including confidentiality and voluntary participation, were rigorously followed. Data analysis was performed using JASP version 0.81.3. Descriptive statistics, including frequencies, percentages, means, and standard deviations, were used to summarize the data and identify trends in sustainable design practices and attitudes. To test the hypothesis on the relationship between awareness of sustainable design concepts and actual practices, Pearson's product-moment correlation was employed. The significance level was set at 0.05. This test was selected due to its ability to measure the strength and direction of linear relationships between continuous variables, which aligns with our research question on the impact of awareness on practice. This alignment with the research questions was crucial to understanding the extent to which theoretical knowledge translates into practical application in the field. To test the hypothesis, two variables were identified: (1) awareness of sustainable design concepts, principles, and SDGs in architecture, and (2) the practice of architectural design. Respondents were asked, through a questionnaire, whether their awareness of sustainable design concepts, principles, and SDGs influenced their architectural practice. This question was dichotomous, requiring a 'YES' or ‘NO’ response. For the purpose of the analysis, the “YES” responses, indicating awareness, were considered as the dependent variable, while the “NO” responses were treated as the independent variable. Pearson's Product Moment Correlation Coefficients were used to test the stated hypothesis at a significance level of 0.05. By employing this methodology, the study aimed to determine the extent to which architects' awareness of sustainable design concepts, principles, and SDGs correlated with their actual architectural design practices in the study area. Though some limitations inherent to survey-based research are considered in this current study. Like sample bias, due to the focus on registered firms, potentially excluding freelancers or informal practices. However, those attached to firm may have been accounted for. Also, response bias is possible, as those more interested in sustainability within the organization may have been more likely to respond. The results of this analysis would provide insights into the relationship between theoretical knowledge and practical application of sustainable design principles in the field of architecture. Analysis of tables and charts visually represent results for clear interpretation aligned to the study objectives on role evolution in sustainable architectural practice. This rigorous quantitative methodology facilitates generalizable insights into an emergent research priority – contextualizing sustainability integration in Nigerian architecture across practice and pedagogy.

## 4 Results

Out of the 108 architectural firms sampled for this study, 96 returned completed questionnaires, yielding a high response rate of 88.9 %. This robust response rate enhances the reliability and representativeness of the study findings. The high participation level suggests a strong interest in the topic of sustainable architecture among professionals in the study area.

This first section of the questionnaire sought to identify Socio-demographic characteristics of respondent and practice details to access their suitability for the survey. Table 1 illustrates details of respondents Socio-demographic characteristics.

**Table 1:** Socio-demographic characteristics of respondents (n = 96).

Characteristics	Frequency	Percent (%)
**Gender**		
Male	82	85.4
Female	14	14.6
**Age Groups (years)**		
20 years and below	0	0
21–40 years	10	10.4
41–60 years	62	64.6
>60 years	24	25
**Level of Education**		
HND/B.Sc.	0	0
M.Sc.	80	83
PHD	16	17
**Scale of Operation**		
Small	5	5.2
Medium	53	55.2
Large	38	39.6
**Years of Practice**		
0–5 years	9	9.4
6–10 years	23	24.0
11–20 years	27	28.1
20 years and above	37	38.5

This sample profiling reveals a dominance of highly experienced male professionals – 85.4 % men and 64.6 % aged 41–60 years. This can be attribute to prevalence of male gender in the building and construction sector. With over a third practicing for over 20 years, extensive industry exposure facilitates nuanced perspectives. The 83 % postgraduation rate also signifies elevated educational capabilities because the licensure criteria require you obtain a professional master's degree. Regarding firm scale dynamics, while 55.2 % medium entities dominate, 5.2 % small alongside 39.6 % large organizations prevents skew. Varied workplace sizes prevent narrow lenses. The level of professional maturity among respondents reflects a thorough understanding of both building and design expertise, construction protocols and other collaboration subtleties undergirding construction initiatives.

The second section of the questionnaire explored respondents' perspectives on architects' implementation of sustainable design principles, covering their awareness, extent of application, influencing factors, and perception of integration into architectural education in the study area. Table 2 provides details of respondent's awareness of sustainability in architectural design.

**Table 2:** Awareness of respondent's perception of sustainability in architectural design**.**

Variable	Percentage response	
	Yes	No
Sustainability Concepts	100	0
Sustainable design principles	96	4
SDGs in architecture	70	30

From Table 2, there is a positive finding that 100 % of the 96 surveyed architects self-reported having awareness of sustainability concept, implying a general understanding of various pillars of sustainability.

**Fig. 3:**
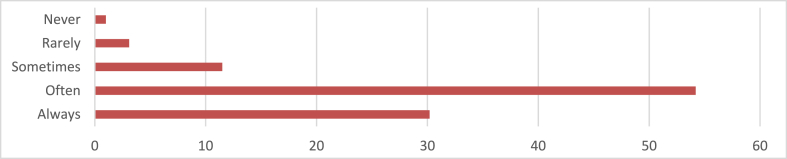
Incorporation of Sustainable design principles in architectural projects.

From Fig. 3, the majority of respondents (32.2 %) consistently incorporate sustainable design principles in their architectural projects, with 54.2 % choosing often. However, 11.5 % choose sometimes, indicating potential barriers to consistent integration. Only 3.1 % rarely incorporate sustainable principles, and one person opts never, highlighting the importance of understanding the reasons behind their choices for the architectural profession and the environment.

**Fig. 4:**
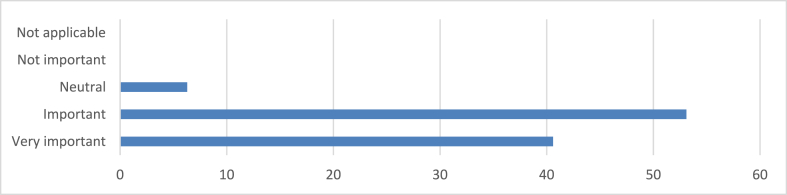
Resource efficiency in sustainable architectural designs.

As seen in Fig. 4, most respondents (40.6 %) consider resource efficiency crucial in sustainable architectural designs, indicating a strong commitment to responsible use and material conservation. The majority (53.1 %) also view resource efficiency as important, indicating a consensus on its high value. A small percentage (6.3 %) expressed a neutral opinion, indicating a unanimous acknowledgment of resource efficiency's importance.

**Fig. 5:**
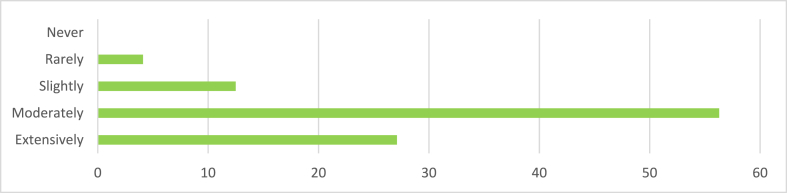
Integration of renewable energy sources in architectural projects.

From Fig. 5, 27.1 % of respondents integrated renewable energy sources extensively into their architectural projects, demonstrating a proactive approach to sustainability. The majority (56.3 %) reported a moderate level of integration, while 12.5 % had only a slight integration and 4.1 % rarely integrated. The fact that none of the respondents said they had never included renewable energy sources shows how well accepted this concept is in architectural designs.

**Fig. 6:**
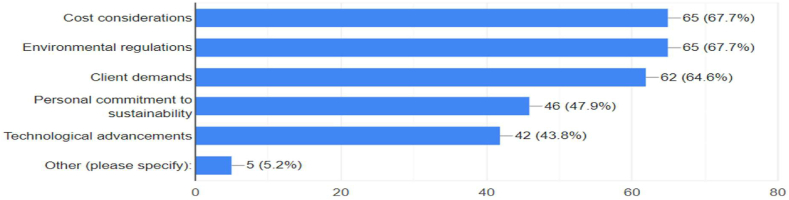
Factors that influence adaptation to sustainable architectural practices.

The survey results depicted in Fig. 6 show that 67.7 % of respondents prioritize cost considerations and compliance with environmental regulations, indicating the significant influence of economic factors and regulations on sustainable practices adoption. Additionally, 64.6 % highlight clients' demand as a key factor, reflecting the strong influence of client preferences. About 47.9 % emphasize personal commitment to sustainability, while 43.8 % recognize technological advancement as crucial, showcasing the role of sustainable technologies.

**Fig. 7:**
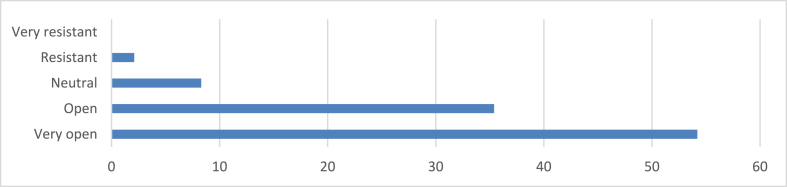
Openness to changes in design methodologies.

The survey findings revealed in Fig. 7 evidenced that 54.2 % of respondents express a high level of openness to changes in design methodologies for sustainable practices, indicating a positive inclination towards embracing new approaches. Additionally, 35.4 % show openness towards such changes, reflecting a favorable attitude towards sustainability integration. Only 8.3 % provide a neutral response, while 2.1 % report rarely being open to changes, indicating some resistance. Notably, no respondents indicate being very resistant to changes, suggesting overall openness within the surveyed group.

**Fig. 8:**
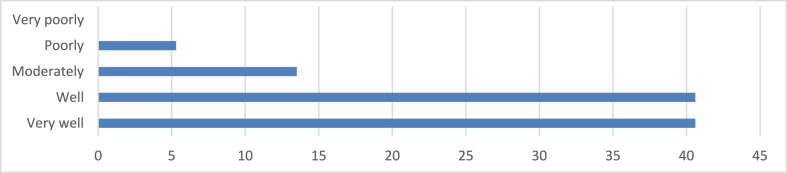
Integration of sustainable design into architectural education.

Fig. 8 results indicate that 40.6 % of respondents believe sustainable design is integrated very well into architectural education, reflecting a positive perception of the curriculum's effectiveness. Similarly, an equal number (40.6 %) express satisfaction with the current level of integration, reinforcing the positive sentiment. A smaller group (13.5 %) perceives the integration as moderate, indicating a more cautious stance with potential for improvement. Additionally, 5.3 % express dissatisfaction, highlighting a perceived gap in the integration of sustainable principles. Notably, no respondents indicate extreme dissatisfaction, suggesting overall contentment despite some concerns.

**Fig. 9:**
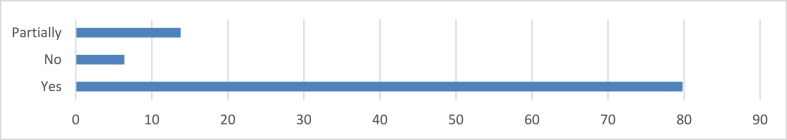
Education on sustainable design principles during architectural studies.

The survey data in Fig. 9, it reveals that 79.8 % of respondents feel they have received adequate training or education on sustainable design principles during their architectural studies, indicating a positive perception of their preparedness in this area. Conversely, a small proportion (6.4 %) report not receiving sufficient training, suggesting a potential gap in their educational experience. Additionally, 13.8 % mention receiving partial training, highlighting a mixed experience with opportunities for improvement in the comprehensiveness of sustainable design education.

**Fig. 10:**
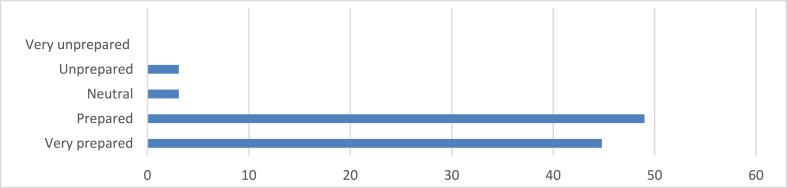
Level of preparedness to address challenges and opportunities in sustainable practice.

From Fig. 10, a significant proportion of respondents (44.8 %) express feeling very prepared to address challenges and opportunities in sustainable practice based on their education, indicating a strong sense of confidence among this group. Additionally, a substantial number (49 %) report feeling prepared, further underscoring the overall positive perception of readiness within the surveyed population. However, a small percentage (3.1 %) provide a neutral response, indicating ambivalence towards their preparedness, while another 3.1 % express feeling unprepared, suggesting a potential gap in their perceived readiness for sustainable practice. Notably, no respondents indicate feeling very unprepared, indicating that while some may feel unprepared, extreme lack of confidence is absent.

### 4.1 Analytical result of the influence sustainability awareness on their architectural design practices

The percentage perceptions of the respondents on the influence of architects' awareness of sustainable design concepts, principles, and Sustainable Development Goals (SDGs) on their architectural design practices in the Enugu region are in Table 3. The findings showed variation in the respondent's perception, which may be linked to personal observation, choice, or client preference.

**Table 3:** Result summary of Pearson product moment correlation coefficient.

			n	Pearson's r	p
YES	–	NO	3	−1.000	<0.001

From the analysis of the significant relationship between awareness of sustainable design concepts, principles, and Sustainable Development Goals (SDGs) and architectural practice, as shown in Table 3, the computed result of the Pearson's Product Moment Correlation Coefficient between the predictor X (YES) and Y (NO) revealed a P-value of 0.001. Since the P value [0.001] is less than the 0.05 level of significance, “Ho” is rejected. The researchers, therefore, conclude that there is a significant relationship between awareness of sustainable design concepts, principles, and Sustainable Development Goals (SDGs) on the architectural practice in the region. This positive relationship indicates that an increase in awareness is associated with an enhancement of sustainable architectural practice. The positive nature of this relationship suggests that each unit increase in awareness corresponds to almost a unit increase in the preservation and promotion of sustainable architectural design in the Enugu region. On the other hand, the result implies that the inadequate awareness in the Enugu region can potentially devalue the sustainable architectural design practice that fosters sustainable development.

This positive correlation can be explained through the lens of the review underpinning theories.

• The **Diffusion of Innovation Theory** suggests that as awareness (an innovation) spreads through the architectural community, early adopters demonstrate the benefits, leading to wider acceptance and integration of sustainable practices. The stages of diffusion—knowledge, persuasion, decision, implementation, and confirmation—are reflected in the growing awareness and its impact on practice.
• The **Theory of Planned Behaviour** posits that increased awareness positively influences architects' attitudes towards sustainable practices, reinforces supportive subjective norms, and enhances perceived behavioural control. As architects become more knowledgeable about sustainability, they are more likely to view sustainable practices favourably, feel a sense of social obligation to adopt them, and believe they can effectively implement these practices.
• **Ecological Modernization Theory** supports the idea that increased awareness leads to innovation and economic growth. As architects learn more about sustainable practices, they can drive technological advancements and create new markets for sustainable materials and methods, reinforcing the economic and environmental dimensions of sustainability.
• **Social Practice Theory** highlights that as awareness increases, sustainable practices become part of the routines and norms within architectural firms. The interplay between new sustainable materials, the competence to use them, and the evolving meaning of sustainability in professional practice stabilizes these practices over time.
• **Transition Theory** frames this positive correlation as part of a broader socio-technical transition. Increased awareness among architects represents a niche innovation that, through sustained efforts and broader systemic support, can transform the socio-technical regime of architectural practice towards sustainability.

## 5 Discussion

The results of the current study offer valuable insights into the state of sustainable architectural practices in Enugu, Nigeria, highlighting both the challenges and opportunities encountered by architects in third world economies. This discussion contextualizes these results within the broader framework of Nigeria's development challenges and compares them with trends in other developing nations.

### 5.1 Practices and commitment of architects toward incorporating sustainable design strategies

The study's findings unveil a multifaceted landscape of sustainable architectural practices in the study area, reflecting both global trends and local challenges. The high level of awareness among architects regarding sustainability concepts (100 % of respondents) and sustainable design principles (96 % of respondents) aligns with the global shift towards environmentally conscious architecture. This awareness forms a crucial foundation for the implementation of sustainable practices, as posited by the Diffusion of Innovation Theory [49]. According to this theory, awareness is the first stage in the adoption of new practices, followed by persuasion, decision, implementation, and confirmation. This finding on awareness levels is consistent with those reported by Ref. [27] in their study of architectural students at Kaduna State University, which found moderate to high levels of awareness about sustainable building practices. However, Ibiyeye et al. [27] noted that this awareness was primarily theoretical, with limited practical application - a gap that our study also identifies among practicing architects in Enugu. The translation of awareness into consistent practice reveals a more nuanced picture. While 32.2 % of respondents report consistently incorporating sustainable design principles in their projects, and 54.2 % do so often, there remains a gap between knowledge and implementation. This discrepancy can be understood through the lens of the Theory of Planned Behaviour, which suggests that the intention to adopt sustainable practices is influenced not only by attitudes but also by subjective norms and perceived behavioural control. The gap between awareness and implementation is not unique to Nigeria. A study by Marsh et al. [62] in South Africa found similar challenges, identifying barriers such as lack of client demand, higher initial costs, and limited availability of green materials. These factors likely contribute to the fact that 11.5 % of architects in our study only sometimes incorporate sustainable principles, and 3.1 % rarely do so.

The integration of specific sustainable strategies, such as resource efficiency and renewable energy sources, presents a mixed picture. The high value placed on resource efficiency (93.7 % of respondents considering it crucial or important) demonstrates a strong commitment to one of the fundamental principles of sustainable architecture. This aligns with international trends identified by Cole [63], who found that water efficiency, waste reduction, and energy conservation are highly prioritized globally. The adoption of renewable energy sources in architectural projects, while promising, shows room for growth. With 27.1 % of respondents extensively integrating renewable energy and 56.3 % doing so moderately, there is a clear trend towards embracing these technologies. This trend resonates with the findings of Smith et al. [64], which ranked renewable energy integration as a top applied strategy among architects globally. However, the fact that 16.6 % of respondents only slightly or rarely integrate these sources indicates persistent barriers, possibly related to cost, technical knowledge, or local infrastructure limitations. These findings can be contextualized within the broader challenges facing sustainable architecture in developing countries. As highlighted by Toriola-Coker et al. [17], sustainability remains an “evolving culture” within Nigerian architecture, requiring further research and policy support for widespread implementation. Their study identified barriers such as lack of awareness, high initial costs, and inadequate government policies - factors that likely contribute to the uneven adoption levels observed in this present study.

The positive trend in sustainable practice adoption among Nigerian architects, with 86.4 % either consistently or often incorporating sustainable principles, is encouraging. However, it's important to note that self-reported data may overestimate actual adoption levels, as pointed out by Ref. [65] in their global survey analysis. This discrepancy underscores the need for observational studies and post-occupancy evaluations to accurately assess the real-world implementation of sustainable practices. The findings on the integration of renewable energy sources are particularly relevant given Nigeria's energy challenges. Obi et al. [66] noted substantial interest in solar technologies among Nigerian architects, which aligns with our results. However, they emphasized the need for greater adoption of holistic techniques going beyond singular solutions - a recommendation supported by our findings on the varied levels of renewable energy integration. The challenges facing sustainable architecture in Enugu mirror broader issues in Nigeria's built environment sector. As the city rapidly expands to accommodate rural-urban migration, the pressure to quickly deliver housing and infrastructure often overshadows sustainability considerations. This conflict between immediate development needs and long-term environmental goals is a common challenge in developing countries, as noted by Saha et al. [67] in their study of barriers to green building adoption in India.

The growing adoption of sustainable practices among Enugu's architects exemplifies the principles of Ecological Modernization Theory, representing part of a broader shift towards integrating environmental concerns into economic development. This theory posits that environmental protection and economic growth can be mutually reinforcing, suggesting that the transition to sustainable architecture could drive innovation and create new economic opportunities in Nigeria's construction sector. The current research result also resonates with those of Opoku and Ahmed [26], who studied sustainability practices in UK construction organizations. While their context differs, they similarly found that awareness and attitudes towards sustainability were generally positive, but implementation was hampered by factors such as cost, lack of client demand, and inadequate regulations. This suggests that some challenges in adopting sustainable practices are common across different contexts, albeit with varying intensities. The moderate integration of renewable energy sources is particularly significant given Nigeria's energy challenges and climate vulnerabilities. As the country faces increasing climate-related risks such as flooding and heat waves, the role of architects in designing resilient, energy-efficient buildings becomes crucial. This aligns with the findings of [32], who emphasized the need for architectural responses to climate change in sub-Saharan African cities.

### 5.2 Factors that motivate adaptation of design methodologies embracing sustainable architecture

The adoption of sustainable architectural practices is driven by a complex interplay of factors that vary across different contexts. The study results reveal that economic considerations, regulatory compliance, client demands, personal commitment, and technological advancements are key factors influencing architects' decisions in adopting sustainable architectural principles in Enugu, Nigeria. These findings align with global trends identified by Ashour et al. [68], who highlighted evolving regulations, client priorities, technological growth, and personal values as top drivers toward sustainability worldwide. Economic considerations and regulatory compliance emerge as primary motivators, with 67.7 % of respondents citing these as significant factors. This high percentage underscores the importance of financial viability and legal requirements in driving sustainable practices. The emphasis on economic factors aligns with the Ecological Modernization Theory [51], which posits that environmental protection and economic growth can be mutually reinforcing. In the context of Enugu's architectural landscape, this suggests that sustainable practices are increasingly seen as economically viable and necessary for compliance with evolving regulations. Client demand also plays a crucial role, with 64.6 % of respondents identifying it as a key factor. This finding resonates with global trends reported by AlSanad [69], who noted that over 75 % of construction firms worldwide are experiencing increased sustainability-related queries from clients. The significant influence of client preferences demonstrates key principles of the Theory of Planned Behavior were societal norms and perceived benefits shape individual and organizational decisions. As clients become more environmentally conscious, their demands drive architects to incorporate sustainable design principles. Personal commitment to sustainability emerges as another important factor, with 47.9 % of respondents emphasizing its significance. This internal motivation aligns with the concept of value-belief-norm theory, suggesting that personal values and beliefs about environmental responsibility influence professional practices. The relatively high percentage of architects citing personal commitment indicates a growing ethical consciousness within the profession regarding environmental sustainability. Technological advancements are recognized as a crucial driver by 43.8 % of respondents. This acknowledgment of technology's role in facilitating sustainable design practices aligns with the findings of Yu et al. [70], who evaluated how innovations like renewable materials, green roofs, and optimization software are removing adoption barriers. The integration of these technologies into architectural practice can be understood through the Diffusion of Innovation Theory, where the perceived benefits and ease of use of new technologies drive their adoption.

The study also reveals a high level of openness to change among architects in Enugu, with 89 % of respondents indicating willingness to adapt their design methodologies. This openness is crucial for the adoption of sustainable practices and aligns with findings from Diugwu et al. [71], who noted that over 62 % of Nigerian architects reported high openness to change. This willingness to adapt is essential for the diffusion of sustainable practices within the architectural community. The factors motivating sustainable design adoption in Enugu echo findings from studies in other developing countries. Research in India by Ref. [67] and in South Africa by Ref. [62] similarly found that cost concerns and market demand were primary drivers for green building practices. This suggests common challenges and opportunities across developing nations in transitioning to sustainable architecture. However, the study also highlights the need for context-specific considerations. As Borsos et al. [72] discussed, more research is essential regarding contextual factors like culture, climate, and locale-specific hindrances in Nigeria. This represents wider African challenges in pairing sustainability goals with regional solutions. The unique climatic conditions of Enugu, for instance, necessitate tailored approaches to passive cooling and energy efficiency that may differ from global standards. The high level of openness to change reported by architects presents an opportunity to align sustainable building practices with Nigeria's economic diversification goals. By fostering a local green building industry, including manufacturing of sustainable materials and technologies, the architectural sector could contribute to job creation and economic growth while addressing environmental challenges. This potential aligns with the principles of Ecological Modernization Theory, suggesting that environmental protection and economic development can be mutually beneficial. However, realizing this potential requires addressing several barriers, particularly around costs and regulatory frameworks. The emphasis on economic considerations in the study results suggests that financial incentives and supportive policies could play a crucial role in accelerating the adoption of sustainable practices. Furthermore, the significant influence of regulatory compliance indicates that strengthening and enforcing sustainability-focused building codes could be an effective strategy for promoting sustainable architecture in Enugu.

### 5.3 Integration of sustainability education within architectural curricula for future architects

The integration of sustainability principles into architectural education plays a critical role in defining the future of sustainable design practices. A significant proportion of respondents (40.6 %) believe that sustainable design is integrated very well into architectural education, reflecting a positive perception of the curriculum's effectiveness. The obtained results resonate harmoniously with existing literature, underscoring the persistent need to augment sustainable design education within the field of architecture. The importance of sustainable design education has been consistently highlighted in studies such as those conducted by Refs. [73,74]. Johnson and Brown [73] emphasized the significance of integrating sustainability into architectural education, fostering a foundation for environmentally conscious practices among emerging architects. Similarly, Greenfield et al. [74] conducted a comparative analysis, reinforcing the crucial role of education in shaping sustainable architectural practices. The congruence between these findings and established literature underscores a consensus within the architectural community regarding the ongoing necessity to enhance educational efforts. This alignment affirms the importance of continuous improvement in sustainable design education to empower future architects with the knowledge and skills needed for environmentally responsible practice. On the global perspective, this finding aligns with trends observed by Ref. [75], who found that over 80 % of architectural programs in the UK and USA offer dedicated sustainability courses. The positive perception in Enugu suggests that local educational institutions are making concerted efforts to incorporate sustainability into their curricula, recognizing its growing importance in the field of architecture. However, the study reveals varying levels of satisfaction with sustainability integration in architectural education. While 40.6 % of respondents express satisfaction with the current level of integration, 13.5 % perceive it as moderate, indicating room for improvement. This variation in perceptions aligns with findings from Seyis and Ergen [76], who identified perceptible skill mismatches between perceived and actual sustainable architectural capabilities among graduates. The discrepancy suggests that while sustainability is being addressed in educational programs, there may be gaps between theoretical knowledge and practical application. The study's findings on education align closely with the principles of the Theory of Planned Behavior. This theory posits that individuals' intentions to engage in specific behaviors (in this case, sustainable architectural practices) are influenced by their attitudes, subjective norms, and perceived behavioral control. Educational initiatives are instrumental in influencing factors, particularly in developing positive attitudes towards sustainability and increasing perceived behavioral control through knowledge and skill development. Encouragingly, 79.8 % of respondents report receiving adequate training or education on sustainable design principles during their architectural studies. This high percentage suggests that sustainability has become a significant component of architectural education in Enugu. However, the fact that 6.4 % report not receiving sufficient training, and 13.8 % mention receiving only partial training, highlights ongoing challenges in ensuring comprehensive sustainability education for all students. These findings resonate with the work of Asiabo and Adekunle [77], who identified self-efficacy versus competency gaps in core sustainability areas among Nigerian architecture students. The discrepancy between perceived adequacy of training and actual competency levels underscores the need for continued refinement and enhancement of sustainability education in architectural programs.

The study also sheds light on architects' preparedness to address challenges and opportunities in sustainable practice. A significant proportion (44.8 %) express feeling very prepared, with an additional 49 % feeling prepared. This high level of perceived readiness is encouraging and suggests that educational programs are instilling confidence in graduates regarding their ability to implement sustainable practices. However, the small percentage (3.1 %) feeling unprepared indicates that there is still work to be done in ensuring all graduates feel equipped to tackle sustainability challenges. These findings can be interpreted through the framework of Social Practice Theory, which emphasizes the importance of skills, materials, and meanings in shaping practices. Educational programs have a fundamental role in developing the skills and knowledge (competences) needed for sustainable architecture, as well as shaping the meanings associated with sustainability in architectural practice. The high levels of perceived preparedness suggest that education is successfully contributing to the development of these elements.

However, the study also highlights the need for more practical, hands-on training in sustainable design techniques. This aligns with global trends towards experiential learning in architectural education, as noted by Yazıcıoğlu and Sarpaşar [78]. Practical experience is crucial for bridging the gap between theoretical knowledge and real-world application, ensuring that graduates are truly prepared to implement sustainable practices in their professional careers. The findings also highlight the importance of continuous professional development in the rapidly evolving field of sustainable architecture. As noted by Ref. [79], pedagogical updating aligned to regional needs remains vital for holistic integration of sustainability principles, particularly in developing economies facing resource constraints. The integration of sustainability into architectural education in Enugu can be seen as part of a broader transition towards sustainable practices, as conceptualized in Transition Theory. Educational institutions play a crucial role in this transition by nurturing niche innovations (sustainable design practices) that can eventually transform the broader socio-technical regime of architectural practice.

## 6 Conclusion and recommendations

This research pursued issues pertaining to sustainable architecture in having Enugu city, South-east Nigeria as its scope and area of study. This study investigates the evolving integration of sustainability considerations among architects in Enugu Metropolis, Nigeria, garnering empirical perspectives on awareness, adoption factors, and educational preparedness. The findings reveal high levels of sustainability concept awareness and openness towards principle assimilation in architectural projects. However, uneven integration persistence calls for reinforcing adoption drivers like compliant codes, client demands and supportive technologies. Additionally, architectural education alignments require further strengthening to propagate sustainability across core design studios for competency development. While positive sustainability orientations exist, mainstreaming green building knowledge and practices remains gradual amidst pressing climate change challenges. Recommendations form this current study includes;

• Update architectural curricula and accreditation mandates to integrate sustainability comprehensively, emphasizing applied building techniques instead of conceptual foundations alone.
• Boost climate consciousness from initiation by mandating green ratings for public projects and upgrading building codes to promote energy efficiency, renewable adoption etc.
• Incentivize developers and homeowners to demand sustainability features through promotional schemes, tax rebates and low-cost financing to elevate market pull factors.
• Encourage interdisciplinary collaborations, industry mentorships and global best practice knowledge transfer to accelerate localized sustainability solution development.
• Propagate specialized sustainability certification and continuous professional education to elevate and update capabilities.
• Commission further research quantifying post-occupancy impacts, user perspectives and field applications to strengthen empirical insights.

The recommendations provide strategic avenues for education policymakers, regulatory agencies, professional bodies, and architects themselves to collectively transform the trajectory of development practices towards climate-compatible and socially equitable habitat creation. Though sustainability integration remains moderate currently, purposeful efforts addressing identified gaps can position Nigerian architects to lead the sustainability transition while meeting local built environment needs.

### 6.1 Suggestion for further research areas

Future research could address these limitations by incorporating observational studies of actual sustainable design practices, expanding the geographic scope to include other Nigerian cities for comparison, and conducting longitudinal studies to track changes in sustainable design adoption over time. Additionally, investigating the perspectives of clients and end-users of sustainably designed buildings could provide valuable insights into the practical impacts of these practices.

## CRediT authorship contribution statement

**Emeka J. Mba:** Supervision, Methodology. **Francis O. Okeke:** Writing – review & editing, Writing – original draft, Visualization, Project administration, Methodology, Investigation, Formal analysis, Data curation, Conceptualization. **Ajuluchukwu E. Igwe:** Writing – review & editing, Supervision, Resources, Project administration, Investigation. **Chinelo A. Ozigbo:** Methodology. **Peter I. Oforji:** Writing – review & editing, Investigation, Funding acquisition. **Ikechukwu W. Ozigbo:** Writing – original draft, Funding acquisition, Conceptualization.

## Informed consent to participate

The authors confirm they sought and got informed consent from all participants in the study.

## Consent to publish

All named authors in the paper have agreed that the manuscript be submitted for publication.

## Ethical approval

The study protocol was approved by ethic review committee of the department of Architecture in accordance with the ethics guidelines and regulations of the University of Nigeria and followed the Declaration of Helsinki—principles of informed consent, voluntary participation and withdrawal, confidentiality, and privacy of the participants.

## Data availability

The authors confirm that the data supporting the findings of this study are available within the article and its supplementary materials, available at the behest of the first author.

## Funding

This research did not have the courtesy of any funding from any organization.

## Declaration of competing interest

The authors declare that they have no known competing financial interests or personal relationships that could have appeared to influence the work reported in this paper.
